# How will previous infection or current vaccination strategies protect us from future SARS‐CoV‐2 variant infections?

**DOI:** 10.1002/mef2.20

**Published:** 2022-10-17

**Authors:** Olivia Monteiro

**Affiliations:** ^1^ Center for Biomedicine and Innovations, Faculty of Medicine Macau University of Science and Technology Taipa, Macau SAR China

A recent important case–control study by Altarawneh et al.^1^ compared the effectiveness of previous infection and vaccination with homologous series of BNT162b2 or messenger RNA (mRNA)‐1273 on symptomatic Omicron infections in Qatar. This highlight summarises the findings of Altarawneh et al. published in the New England Journal of Medicine.

The SARS‐CoV‐2 Omicron variant has high transmissibility and has become the dominant strain worldwide. Multiple sublineages emerged from the Omicron B.1.1.529 including BA.1, BA.2, BA.3, BA.4, and BA.5 subvariants. As populations are gaining immunity against SARS‐CoV‐2 via natural immunity from previous infections or by vaccination or both, it is important to follow the effectiveness of protection against Omicron variants.

This study has matched polymerase chain reaction (PCR)‐confirmed cases of COVID‐19 to controls with a negative PCR test that were identified from December 23, 2021 to February 21, 2022 in Qatar using data from the whole population since the start of the pandemic. The criteria for matching case participants and controls were sex and age within a 10‐year range. Qatar has a diverse population made up mostly of expatriates (89%) from other countries. Case participants and controls are also matched according to nationality to investigate if one population is differentially affected to another. In addition, the calendar week of PCR test is matched to control for any SARS‐CoV‐2 spreading events occurring within the period of the study. Case participants were individuals who had clinical symptoms of SARS‐CoV‐2 infection and had tested positive in a PCR test. Effectiveness of protection refers to the odds of previous infection or vaccination or both among case participants compared with test‐negative controls. The five groups analysed were: (1) previous infection and no vaccination; (2) two‐dose vaccination and no previous infection; (3) two‐dose vaccination and previous infection; (4) three‐dose vaccination and no previous infection; (5) three‐dose vaccination and previous infection. Participants received homologous series of BNT162b2 or mRNA‐1273. The authors assumed that all previous infections were caused by preomicron variants since these infections occurred before the omicron wave in Qatar. A previous infection was defined as a positive PCR test for SARS‐CoV‐2 at least 90 days before the current positive PCR test. The table below summarises the results.

Figure 1 summarises the results on effectiveness of the different kinds of acquired immunity. Although two‐dose vaccination and no previous infection had negligible effectiveness against BA.1 and BA.2 infections, the authors note that the median interval between the second vaccine dose and the PCR test for BA.1 and BA.2 was 268 and 270 days, >8 months after receiving the first booster vaccine shot. The median time between third dose and PCR test is 42–43 days. Therefore, higher neutralizing antibody titres can be expected from participants who have received the third dose. mRNA‐1273 had very similar effectiveness against BA.1 and BA.2 in all the groups studied compared with BNT162b2. The authors also measured effectiveness of prior infections and vaccination against symptomatic Omicron infection over time, providing an estimation of how long immunity acquired by different means lasts. A previous infection offered 48.3%–51.0% protection against reinfection 10–12 months after, compared to 13.9% and 18.6% 4–6 months after the second BNT162b2 or mRNA‐1273 dose. The primary‐series vaccination, two homologous doses or BNT162b2 or mRNA‐1273, offers short‐lived protection against Omicron variants. This suggests that previous infections offer more durable protection against BA.1 and BA.2 compared to two doses of either mRNA vaccine. Indeed, the rate of infection by the delta variant was found to be significantly increased at 4–6 months after two doses of BNT162b2 compared to an unvaccinated cohort who has recovered from a previous infection 4–6 months prior.^2^ Unsurprisingly, a previous infection plus three doses of vaccine was the most effective against any symptomatic BA.1 or BA.2 infection compared to matched individuals with no previous infection and no vaccination. Importantly, previous infection alone, BNT162b2 or mRNA‐1273 vaccination (two or three doses) or hybrid immunity all showed >70% effectiveness against severe, critical or fatal COVID‐19 due to BA.1 or BA.2 infection.

**Figure 1:**
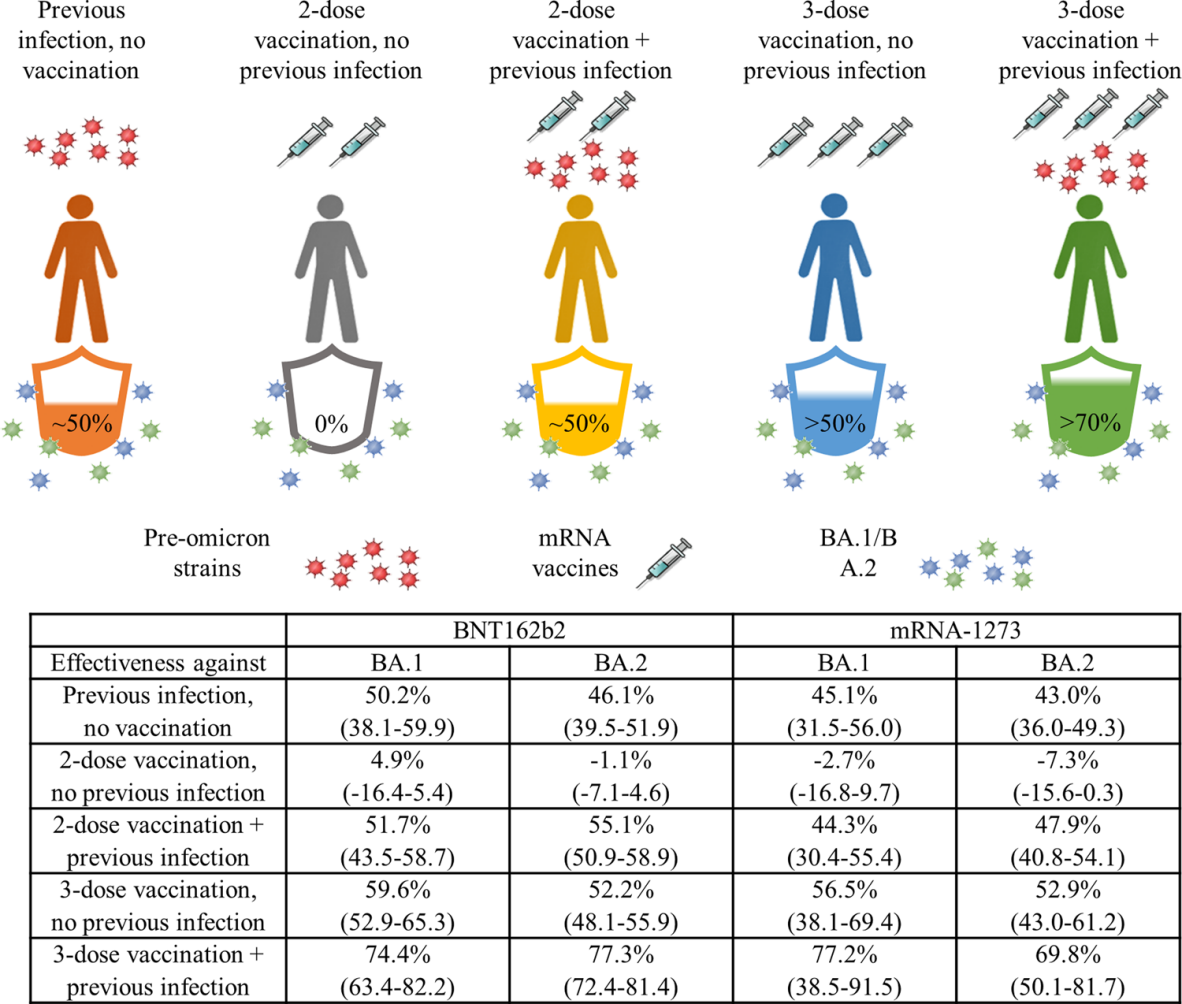
Previous infection with preomicron SARS‐CoV‐2 strains without vaccination provides similar effectiveness against symptomatic BA.1 and BA.2 infection as two‐dose vaccination and preomicron infection. Hybrid immunity with a recent booster vaccination shot confers the strongest protection. Preomicron infection, vaccination alone and hybrid immunity all showed strong effectiveness against severe, critical or fatal COVID‐19. The table summarises the effectiveness of natural immunity acquired from infection with a preomicron SARS‐CoV‐2 strain, BNT162b2, or mRNA‐1273 vaccination without prior infection, or vaccination with previous infection against BA.1 or BA.2. The 95% confidence interval is provided in brackets.

Given the fast pace of SARS‐CoV‐2 evolution, it is important to consider how previous infection and/or vaccination can protect us from newer variant strains. The spike protein of Omicron contains multiple mutations, including those found on the receptor‐binding domain and N‐terminal domain. In addition to common mutations shared between BA.1 and BA.2; BA.1 has 13 mutations that BA.2 lacks, while BA.2 has an additional 8 unique mutations that were not found in BA.1.^3^ In particular, BA.2 lacks the G496S mutation in BA.1 resulting in increased binding affinity of BA.2 to human ACE2 receptors.^4^ Importantly, new data has shown that postvaccination BA.1 infection enriches antibodies that do not compete for binding with ACE2.^4^ These antibodies were also found to be sensitive to mutation at residues 452 and 486, additional mutations found in BA.4 and BA.5,^3^ suggesting prior BA.1 infection may not effectively protect against BA.4 and BA.5 which also lacks the G496S mutation. Omicron variants can evade most WT RBD‐stimulated antibodies, indicating high probability of neutralization escape from vaccine‐induced antibodies from currently available vaccines. Indeed, BA.2.12.1, BA.4, and BA.5, the three most dominant strains in recent months, were more resistant to sera from BNT162b2 or mRNA‐1273 vaccinated and third dose boosted individuals than BA.2.^5^ Neutralizing antibody pseudovirus titer measured from individuals who have recovered from a BA.1/2 infection showed lower neutralizing antibody titers against BA.2.12.1 and BA.4/5 compared to antibody titers against BA.1 and BA.2.^6^, ^7^ Sera from individuals who have received mRNA vaccines also had lower neutralizing antibody titers against BA.2.12.1 and BA.4/5 compared to BA.1 and BA.2. Nevertheless, the data from Altarawneh et al clearly showed that three‐dose vaccination offers more protection than two doses and that antibodies from infection and/or vaccination prevented severe, critical or fatal COVID‐19 effectively.

Given current data that immunity acquired from vaccination or previous infection wanes over time,^2^, ^8^ it is important that we continue to sustain immunity with booster vaccinations.^9^ Recent epidemiology data found increased neutralization of the BA.2 variant from sera of individuals vaccinated with an inactivated vaccine priming dose and boosted with an mRNA vaccine.^10^ This sheds light on the potential value of heterologous vaccine boosters and future vaccination strategies for effective protection against new variant strains. Recent advances in artificial intelligence approaches can be used to predict immune evasion by future variant strains which can inform future vaccine and therapeutics development.

## AUTHOR CONTRIBUTIONS

**Olivia Monteiro**: Conceptualization (lead); Investigation (lead); Resources (lead); Visualization (lead); Writing – original draft (lead); Writing – review and editing (lead).

## CONFLICT OF INTEREST

The author declares no conflict of interest.

### ETHICS STATEMENT

Not applicable.

## Data Availability

Not applicable.
